# Multi-omic analysis reveals elevated BRI3BP expression associated with hepatocellular carcinoma progression and poor prognosis

**DOI:** 10.1038/s41598-025-22072-5

**Published:** 2025-10-31

**Authors:** Ling Liu, Ye Wang, Jintao Zheng, Lixin Zhou, Chenao Yang, Jiachen Zhang, Changku Jia

**Affiliations:** 1 Department of Hepatobiliary and Pancreatic Surgery, Hangzhou First People’s Hospital Affiliated to Medical School of Westlake University, No.261 Huansha Road, Shangcheng District, Hangzhou, 310006 Zhejiang China; 2 Department of Hepatobiliary and Pancreatic Surgery, The Fourth School of Clinical Medicine, Zhejiang Chinese Medical University, No.970 Puyan Road, Binjiang District, Hangzhou, 310053 Zhejiang China; 3 Department of Gastroenterological Surgery, Hangzhou First People’s Hospital Affiliated to Medical School of Westlake University, No.261 Huansha Road, Shangcheng District, Hangzhou, 310006 Zhejiang China; 4 Department of Hepatobiliary and Pancreatic Surgery, The Second Affiliated Hospital, School of Medicine, Zhejiang University, No.88 Jiefang Road, Shangcheng District, Hangzhou, 310009 Zhejiang China

**Keywords:** BRI3BP, Hepatocellular carcinoma, Cuproptosis, Immune infiltration, Prognostic biomarker, Cancer, Computational biology and bioinformatics

## Abstract

BRI3BP, an RNA-binding protein upregulated in multiple cancers, plays an undefined role in hepatocellular carcinoma (HCC). This study investigates its diagnostic and prognostic potential in HCC. Multi-omics analysis of TCGA and GEO data identified BRI3BP-interacting genes and enriched pathways. Genomic and epigenetic alterations were assessed using cBioPortal and MethSurv. Immune infiltration was profiled via ssGSEA and TIMER 2.0. Prognostic relevance was validated through survival analysis, Cox regression, and experimental assays. BRI3BP was overexpressed in HCC and correlated with advanced tumor stage, shorter overall survival (OS), and disease-free survival (DFS). Functional enrichment linked BRI3BP to cell cycle regulation, Rho GTPase activity, and copper homeostasis. Analysis of transcriptomic data revealed that BRI3BP expression also significantly correlated with immune cell infiltration and an immunosuppressive microenvironment, validated by immunohistochemistry showing reduced CD8 + and increased CD68 + cells in high-BRI3BP samples. In vitro, BRI3BP overexpression promoted HCC cell migration and invasion and activated the ROCK signaling pathway, suggesting potential involvement in tumor progression. Drug sensitivity assays confirmed lower lapatinib IC50 in overexpression models. High BRI3BP expression correlates with aggressive HCC phenotypes, poorer survival, and dysregulated oncogenic pathways, supporting its role as a prognostic biomarker and candidate therapeutic target.

**Supplementary Information:**

The online version contains supplementary material available at 10.1038/s41598-025-22072-5.

## Introduction

 Hepatocellular carcinoma (HCC) is a molecularly heterogeneous and difficult-to-treat disease. It accounts for 90% of primary liver cancers and is the third leading cause of cancer-related death worldwide, with over 800,000 new cases each year and a 5-year survival rate of 20%^1^. Chronic liver injury caused by persistent hepatitis B or C virus infection^2^, metabolic dysfunction-associated steatohepatitis (MASLD), or alcohol-related cirrhosis synergistically drives hepatocarcinogenesis through sustained inflammation and dysregulated regeneration^3,4^. Although current multimodal therapies, including surgical resection, radiofrequency ablation, transarterial chemoembolization, and tyrosine kinase inhibitors, provide benefits, HCC remains challenging to treat in three key areas. First, above 50% of early-stage patients experience recurrence within two years after resection due to occult micrometastases^5^. Second, despite recent progress with combination immunotherapy, advanced-stage HCC exhibits marked therapeutic resistance, with objective response rates (ORR) plateauing at 20–30% and only approximately 10% of patients achieving durable survival beyond 24 months^6^. Third, even liver transplant recipients risk graft loss from immunosuppression-driven recurrence. Persistent therapeutic failure, therefore, highlights the urgent need for novel biomarkers that can enable early detection and improve prognostic accuracy.

BRI3BP (BRI3-binding protein), an endoplasmic reticulum (ER)-mitochondria tethering modulator, critically governs cellular homeostasis through its dual role in apoptotic regulation and ER stress adaptive processes, mediated by its ability to orchestrate inter-organellar calcium flux and unfolded protein response (UPR) dynamics^7^. Recent work in cuproptosis, a copper ion-dependent regulated cell death mechanism, reveals unexpected mechanistic links to mitochondrial respiration. Here, excess copper ions directly disrupt lipoylated tricarboxylic acid (TCA) cycle enzymes, triggering proteotoxic stress via the collapse of the sulfur relay system and subsequent disassembly of iron-sulfur clusters^8–10^. In ovarian cancer, cuproptosis regulates the expression of cuproptosis-related genes, which in turn alter the tumor microenvironment, immune cell infiltration, and sensitivity to chemotherapeutic and targeted therapies, ultimately affecting tumor progression and patient prognosis^11^. Notably, elevated BRI3BP expression has been linked to poor prognostic outcomes in several malignancies^12^, suggesting its potential utility as a prognostic biomarker in HCC. Due to its frequent association with aggressive tumors, BRI3BP has become a significant focus of research in cancer studies. Nevertheless, there is a scarcity of in-depth investigations that clarify the connection between BRI3BP and HCC. Therefore, we performed a comprehensive bioinformatics analysis using transcriptomic data. Bioinformatics was used to analyze the differential expression of BRI3BP in HCC. Then, we identified the crucial genes and biological pathways linked to BR3IBP. Furthermore, we investigated the potential association between varying levels of BRI3BP expression and the therapeutic response to drugs.

This research represents the first thorough analysis of BRI3BP expression within HCC. Utilizing bioinformatics techniques, the study explored the predicted pathways, regulatory mechanisms, and the relationship between BRI3BP and immune infiltration in HCC. Experimental validation with qRT-PCR, Western blotting, and functional assays corroborated the role of BRI3BP in HCC progression. These findings may deepen understanding of HCC biology, improve risk stratification, and could be integrated into existing prognostic models, with potential implications for therapeutic strategies.

## Results

### BRI3BP expression and its association with clinical features in HCC

**Fig. 1:**
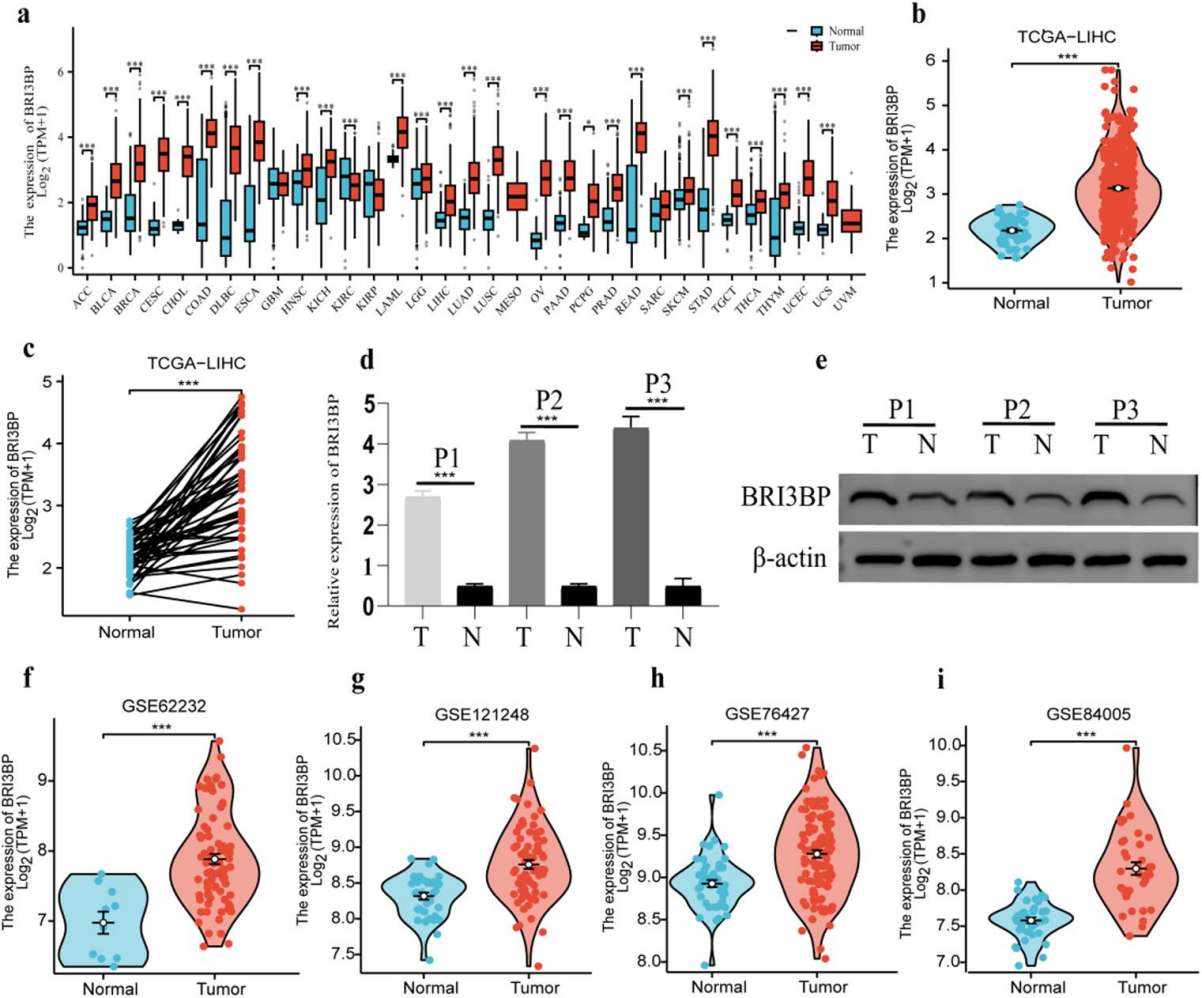
Expression of BRI3BP in various types of tumors. (**a**) Pan-cancer analysis of BRI3BP in TCGA and GTEx databases. (**b**) The expression of BRI3BP between tumor tissues and unpaired normal tissues in TCGA-HCC. (**c**) The expression of BRI3BP between tumor tissues and paired tissues in TCGA-HCC. (**d**) qRT-PCR results about the mRNA expression of BRI3BP in three HCC patients. *BRI3BP* mRNA levels were normalized to *β-Actin*. (**e**) Western blotting result about the protein expression of BRI3BP in three HCC patients. (**f**) GSE62232. (**g**) GSE121248. (**h**) GSE76427. (**i**) GSE84005. ns: *P* ≥ 0.05; *P < 0.05; **P < 0.01; ***P < 0.001.

**Fig. 2:**
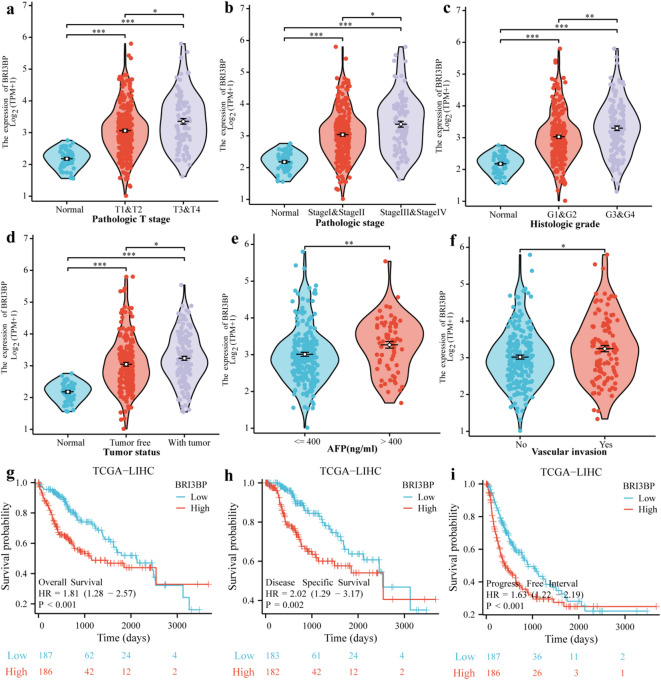
The relationship between BRI3BP and clinical features with prognosis. (**a**) Pathological T stage. (**b**) Pathological stage. (**c**) Histologic grade. (**d**) Tumor status. (**e**) AFP. (**f**) Vascular invasion. (**g**) Overall survival. (**h**) Disease-specific survival. (**i**) Progress Free Interval. ns: *P* ≥ 0.05; *P < 0.05; **P < 0.01; ***P < 0.001.

Pan-cancer multi-omics interrogation of BRI3BP across 28 malignancies spanning epithelial, mesenchymal, and hematopoietic lineages (TCGA PanCancer Atlas) revealed its oncogenic overexpression as a hallmark of aggressive disease biology, including HCC, cholangiocarcinoma (CHOL), kidney renal clear cell carcinoma (KIRC) and lung squamous cell carcinoma (LUSC) (Fig. 1a). Notably, in HCC, BRI3BP expression was markedly elevated in tumor tissues compared to normal hepatic tissues (*P <* 0.001; Fig. 1b). The contrasting expression patterns between tumor and normal tissues are further demonstrated in Fig. 1c, which was also significant (*P* < 0.001). The qRT-PCR and western blotting results indicated that the expression of BRI3BP was significantly higher in HCC tissues compared to adjacent tissues (Fig. 1d and e). Furthermore, validation through the independent transcriptomic datasets GSE62232, GSE121248, GSE76427, and GSE84005 consistently demonstrated the upregulation of BRI3BP in HCC tissues (Fig. 1f and i). In clinical studies, increased BRI3BP expression was found to have a significant correlation with advanced T stage, higher histologic grade, residual tumor status, vascular invasion, and elevated alpha-fetoprotein (AFP) levels (a serum biomarker for HCC), all of which were statistically significant (*P* < 0.05; Fig. 2a and f; as detailed in Table 1). AFP concentration was significantly higher in patients with high BRI3BP expression (*P =* 0.001; Fig. 2e), and vascular invasion was correspondingly more frequent in this cohort (*P* = 0.014; Fig. 2f). Experimental evidence reinforced these observations: BRI3BP overexpression in Huh7 cells increased migration and invasion in Transwell assays, whereas knockdown of BRI3BP in HepG2 cells exerted the opposite effect (Supplementary Fig. S1). Additionally, ectopic BRI3BP expression in HepG2 cells significantly enhanced motility and invasiveness, whereas silencing diminished these properties, as detailed in Supplementary Fig. S2a. Western blotting revealed that BRI3BP overexpression upregulated ROCK1, ROCK2, PDGFB and p-MYPT1, whereas knockdown reduced their abundance (Supplementary Fig. S3), confirming the activation of the ROCK signaling pathway—a recognized driver of HCC progression^13^. Collectively, these data indicate that, in vitro, BRI3BP overexpression is associated with aggressive phenotypes of HCC cells and activates the ROCK signaling pathway, suggesting potential involvement in tumor progression.

**Table 1:** Association of BRI3BP expression levels with clinical characteristics in HCC patients from the TCGA database.

Characteristics	Low expression of BRI3BP	High expression of BRI3BP	*P* value
n	187	187	
Gender, n (%)			0.151
Female	54 (14.4%)	67 (17.9%)	
Male	133 (35.6%)	120 (32.1%)	
Race, n (%)			0.258
Asian	75 (20.7%)	85 (23.5%)	
Black or African American	6 (1.7%)	11 (3%)	
White	98 (27.1%)	87 (24%)	
Age, n (%)			0.070
≤ 60	80 (21.4%)	97 (26%)	
> 60	107 (28.7%)	89 (23.9%)	
Pathologic T stage, n (%)			0.052
T1 & T2	146 (39.4%)	132 (35.6%)	
T3 & T4	38 (10.2%)	55 (14.8%)	
Pathologic N stage, n (%)			0.681
N0	122 (47.3%)	132 (51.2%)	
N1	1 (0.4%)	3 (1.2%)	
Pathologic M stage, n (%)			1.000
M0	131 (48.2%)	137 (50.4%)	
M1	2 (0.7%)	2 (0.7%)	
Pathologic stage, n (%)			0.028
Stage I & Stage II	139 (39.7%)	121 (34.6%)	
Stage III & Stage IV	36 (10.3%)	54 (15.4%)	
Tumor status, n (%)			0.050
Tumor free	111 (31.3%)	91 (25.6%)	
With tumor	68 (19.2%)	85 (23.9%)	
AFP (ng/ml), n (%)			0.001
≤ 400	128 (45.7%)	87 (31.1%)	
> 400	24 (8.6%)	41 (14.6%)	
Histologic grade, n (%)			0.002
G1 & G2	131 (35.5%)	102 (27.6%)	
G3 & G4	54 (14.6%)	82 (22.2%)	
Albumin(g/dl), n (%)			0.888
< 3.5	38 (12.7%)	31 (10.3%)	
≥ 3.5	125 (41.7%)	106 (35.3%)	
Child-Pugh grade, n (%)			0.608
A	122 (50.6%)	97 (40.2%)	
B & C	11 (4.6%)	11 (4.6%)	
Fibrosis Ishak score, n (%)			0.928
0 & 1/2	59 (27.4%)	47 (21.9%)	
3/4 & 5 & 6	60 (27.9%)	49 (22.8%)	
Vascular invasion, n (%)			0.014
No	119 (37.4%)	89 (28%)	
Yes	47 (14.8%)	63 (19.8%)	

### Prognostic and clinical associations of BRI3BP in HCC

**Fig. 3:**
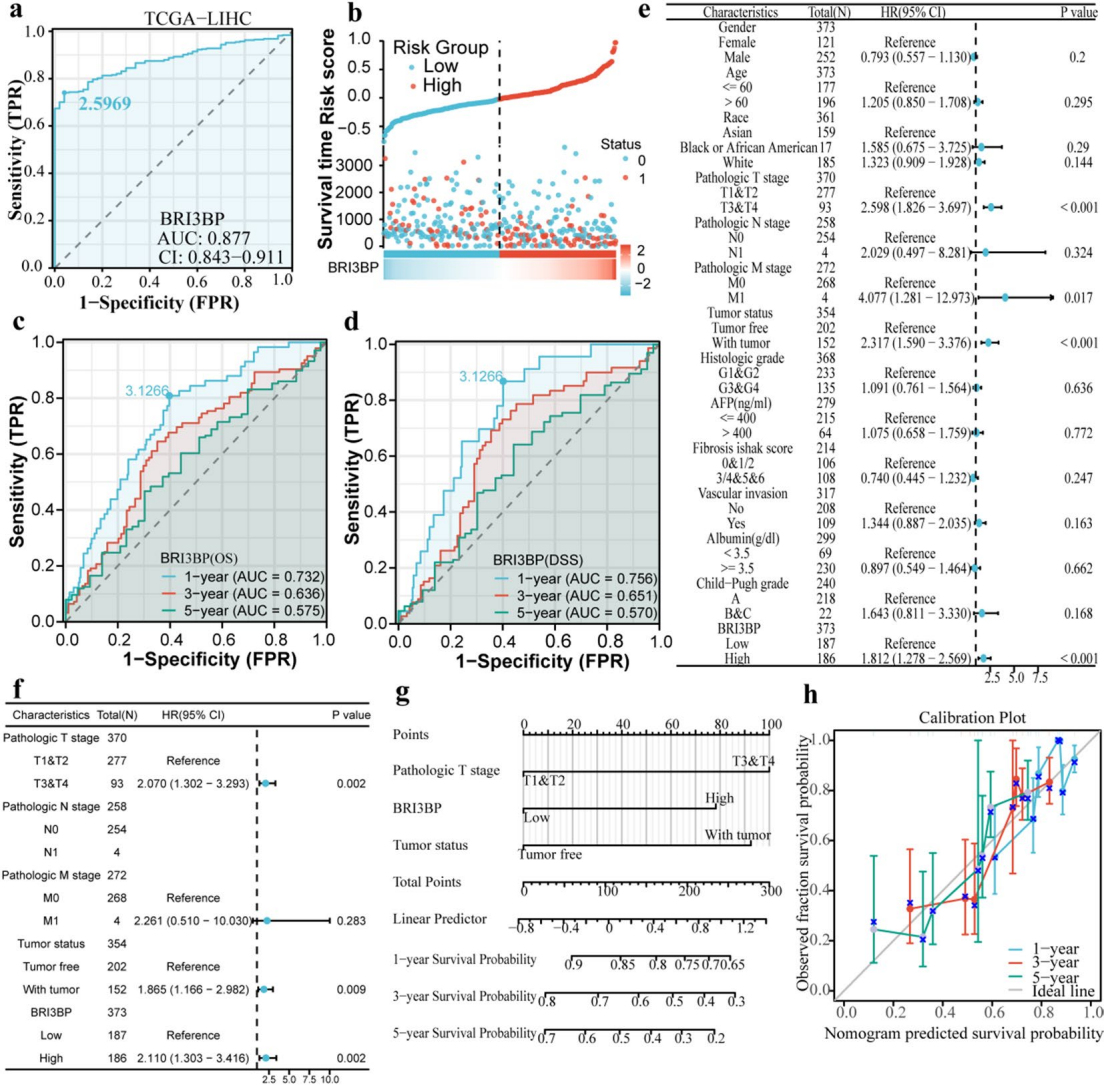
The ROC curve, Cox regression analyses, and nomogram construction of BRI3BP in HCC. (**a**) Diagnostic ROC curves in TCGA. (**b**) Survival time, Risk score and BRI3BP expression heat map in TCGA. (**c**, **d**) Time-dependent ROC curves assessing BRI3BP’s predictive power for OS (**c**) and DSS (**d**) (TCGA). (**e**, **f**) Univariate (**e**) and Multivariate (**f**) Cox regression forest plots. (**g**) Clinically applicable nomogram for predicting 1-, 3-, and 5-year survival. (**h**) Nomogram calibration plots.

To establish BRI3BP as a clinically actionable prognostic determinant in HCC, we analyzed its association with overall survival (OS); time from diagnosis to death from any cause; disease-free survival (DFS); time from treatment to recurrence or death; and progression-free interval (PFI); time from treatment to progression or death (Fig. 2g and i). Kaplan–Meier analysis showed that high BRI3BP expression was linked to poorer OS (HR = 1.81, 95% CI = 1.28–2.57, *P* < 0.001), DFS (HR = 2.02, 95% CI = 1.29–3.17, *P =* 0.002) and PFI (HR = 1.63, 95% CI = 1.22–2.19, *P* < 0.001). Receiver-operating-characteristic (ROC) analysis in the TCGA cohort yielded an area under the curve (AUC = of 0.877), indicating strong discrimination between tumor and adjacent non-tumor tissues (Fig. 3a). A BRI3BP-centred prognostic signature constructed with Cox regression stratified patients into high- and low-risk groups, the high-risk group experiencing recurrence or death significantly sooner (Fig. 3b). Time-dependent ROC curves confirmed the sustained discriminative capacity of BRI3BP for survival outcomes, with 1-year AUCs of 0.732 for OS and 0.756 for DFS, and modest decline at 3 years (OS = 0.636; DFS = 0.651) and 5 years (OS = 0.575; DFS = 0.570) (Fig. 3c, d). Collectively, these findings substantiate the clinical utility of BRI3BP as a prognostic biomarker in HCC.

### Nomogram construction and validation with BRI3BP in HCC

Cox analysis confirmed BRI3BP overexpression as an independent prognostic determinant of diminished OS (HR = 2.110, 95%CI = 1.303 to 3.416, *P* = 0.002) in HCC. Furthermore, Multivariable Cox regression identified advanced T stage (T3-T4 vs. T1-T2) as an independent prognostic determinant of diminished OS (HR = 2.070, 95%CI = 1.302–3.293, *P =* 0.002; as detailed in Table 2). Concurrently, tumor status (non-resected primary lesions) demonstrated a comparable prognostic weight (HR = 1.865, 95%CI = 1.166–2.982, *P* = 0.009; Fig. 3e and f). A multivariable-adjusted prognostic nomogram integrating BRI3BP expression, tumor status, and T stage demonstrated robust discriminative accuracy for 1-, 3-, and 5-year OS prediction (Fig. 3g). The concordance index (C-index) for the full nomogram (BRI3BP + T stage + tumor status) was 0.657 (95% CI: 0.628–0.687). This represented a meaningful improvement over the C-index of the model with T stage and tumor status alone, which was 0.602 (95% CI: 0.579–0.625). Calibration plots revealed minimal deviation between predicted and observed survival probabilities, especially for the 5-year OS, demonstrating the nomogram’s reliability in forecasting long-term survival outcomes (Fig. 3h). Collectively, these results position BRI3BP as a molecularly tractable, stage-agnostic prognostic determinant in HCC.

**Table 2:** Univariate and multivariate analysis of BRI3BP expression.

Characteristics	Total(*N*)	HR (95% CI) Univariate analysis	*P* value	HR (95% CI) Multivariate analysis	*P* value
Pathologic T stage (T3&T4 vs. T1&T2)	370	2.598 (1.826–3.697)	< 0.001	2.070 (1.302–3.293)	0.002
BRI3BP (High vs. Low)	373	1.812 (1.278–2.569)	< 0.001	2.110 (1.303–3.416)	0.002
Pathologic M stage (M1 vs. M0)	272	4.077 (1.281–12.973)	0.017	2.261 (0.510–10.030)	0.283
Tumor status (With tumor vs. Tumor free)	354	2.317 (1.590–3.376)	< 0.001	1.865 (1.166–2.982)	0.009
Pathologic N stage (N1 vs. N0)	258	2.029 (0.497–8.281)	0.324		
Gender (Male vs. Female)	373	0.793 (0.557–1.130)	0.200		
Age (> 60 vs. ≤ 60)	373	1.205 (0.850–1.708)	0.295		
Histologic grade (G3&G4 vs. G1&G2)	368	1.091 (0.761–1.564)	0.636		
AFP(ng/ml) (> 400 vs. ≤ 400)	279	1.075 (0.658–1.759)	0.772		
Fibrosis Ishak score (3/4&5&6 vs. 0&1/2)	214	0.740 (0.445–1.232)	0.247		
Vascular invasion (Yes vs. No)	317	1.344 (0.887–2.035)	0.163		
Race (White vs. Asian)	361	1.323 (0.909–1.928)	0.144		
Albumin (g/dl) (≥ 3.5 vs. < 3.5)	299	0.897 (0.549–1.464)	0.662		
Child-Pugh grade (B&C vs. A)	240	1.643 (0.811–3.330)	0.168		

### Differential gene expression and functional enrichment analysis in HCC

**Fig. 4:**
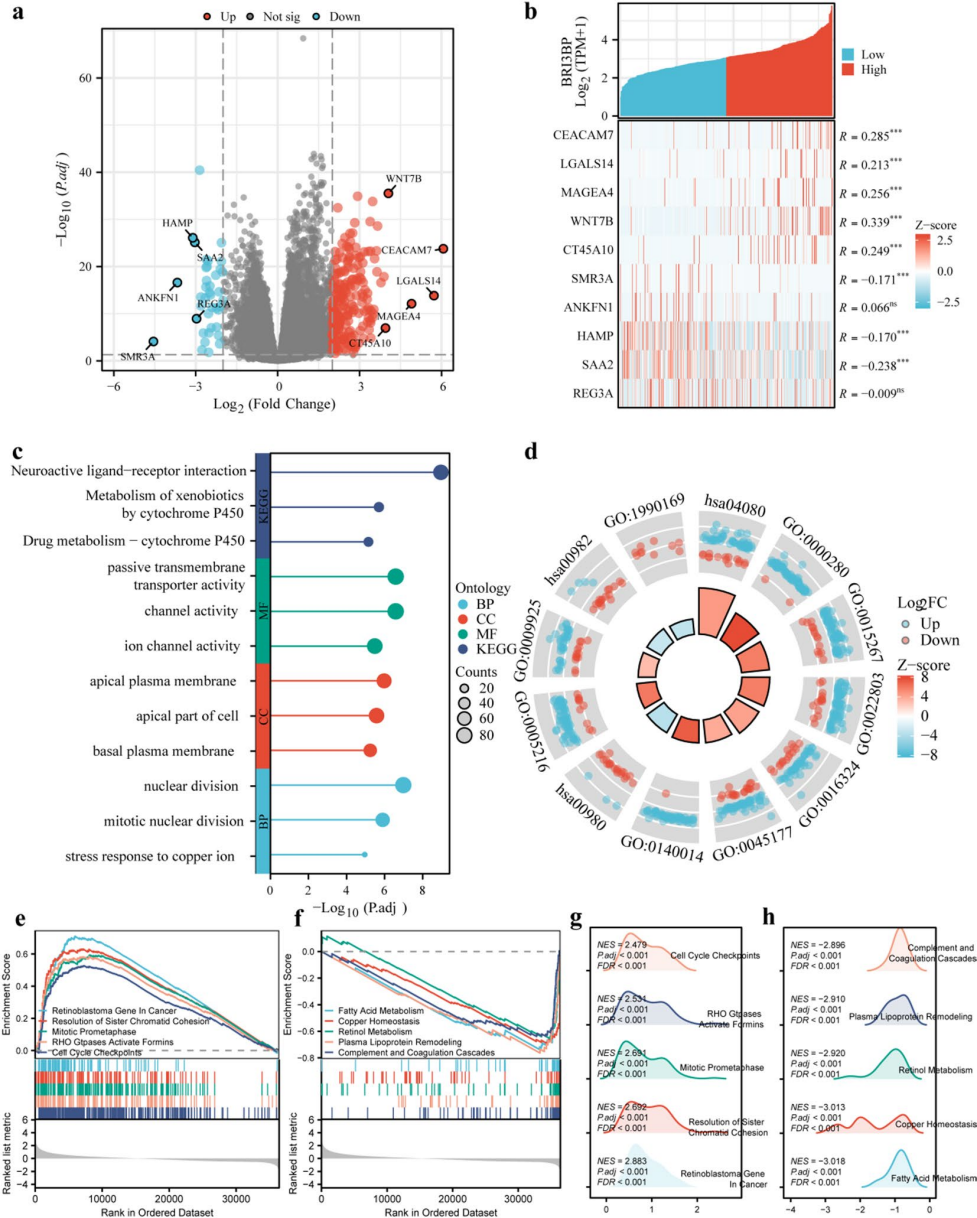
The analysis of DEGs and enrichment plots of BRI3BP in HCC^14–16^. (**a**) Volcano plot of the BRI3BP-related DEGs. (**b**) Heat map of the top five upregulated and downregulated genes with BRI3BP expression. (**c**) Lollipop chart of enrichment analysis. (**d**) Circle diagram (reproduced with permission from Kanehisa Laboratories, www.kegg.jp/kegg/kegg1.html^14–16^. (**e**) GSEA output for BRI3BP-high cohort. (**f**) GSEA output for BRI3BP-low cohort. (**g**) Ridge plot visualization of enriched pathways (BRI3BP-high). (**h**) Ridge plot visualization of enriched pathways (BRI3BP-low).

To dissect the molecular role of BRI3BP, we stratified the TCGA-LIHC cohort into optimally defined high- and low-expression subgroups. A subsequent differential gene expression analysis identified 218 genes that were significantly upregulated and 39 that were notably downregulated (adjusted *P* < 0.05, |log_2_FC| > 2; Fig. 4a). A heatmap analysis of the top five upregulated genes, which included CEACAM7, LGALS14, MAGEA4, WNT7B and CT45A10, alongside the top five downregulated genes (SMR3A, ANKFN1, HAMP, SAA2 and REG3A) provided additional insights into the transcriptional changes associated with BRI3BP expression (Fig. 4b).

We performed functional enrichment analyses to identify the biological processes and pathways influenced by the DEGs associated with BRI3BP. The GO analysis revealed that these DEGs were enriched in pathways linked to channel activity, localization to the apical plasma membrane, and stress responses to copper ions. Furthermore, the KEGG analysis^14–16^ revealed significant pathways impacted by BRI3BP, including neuroactive ligand-receptor interactions and xenobiotic metabolism (Fig. 4c and d). GSEA provided further insights into the biological processes impacted by BRI3BP expression. High BRI3BP expression was positively correlated with various pathways, such as those involving the retinoblastoma gene in cancer, the resolution of sister chromatid cohesion, mitotic prometaphase, Rho GTPase activation of formins, and cell cycle checkpoints. In contrast, low BRI3BP expression was associated with metabolic and regulatory pathways, including fatty acid metabolism, copper homeostasis, retinol metabolism, plasma lipoprotein remodeling, and the complement and coagulation cascades (Fig. 4e and h). These findings implicate BRI3BP in the regulation of both oncogenic and metabolic pathways in HCC.

### Functional analysis of BRI3BP-associated genes and cuproptosis

**Fig. 5:**
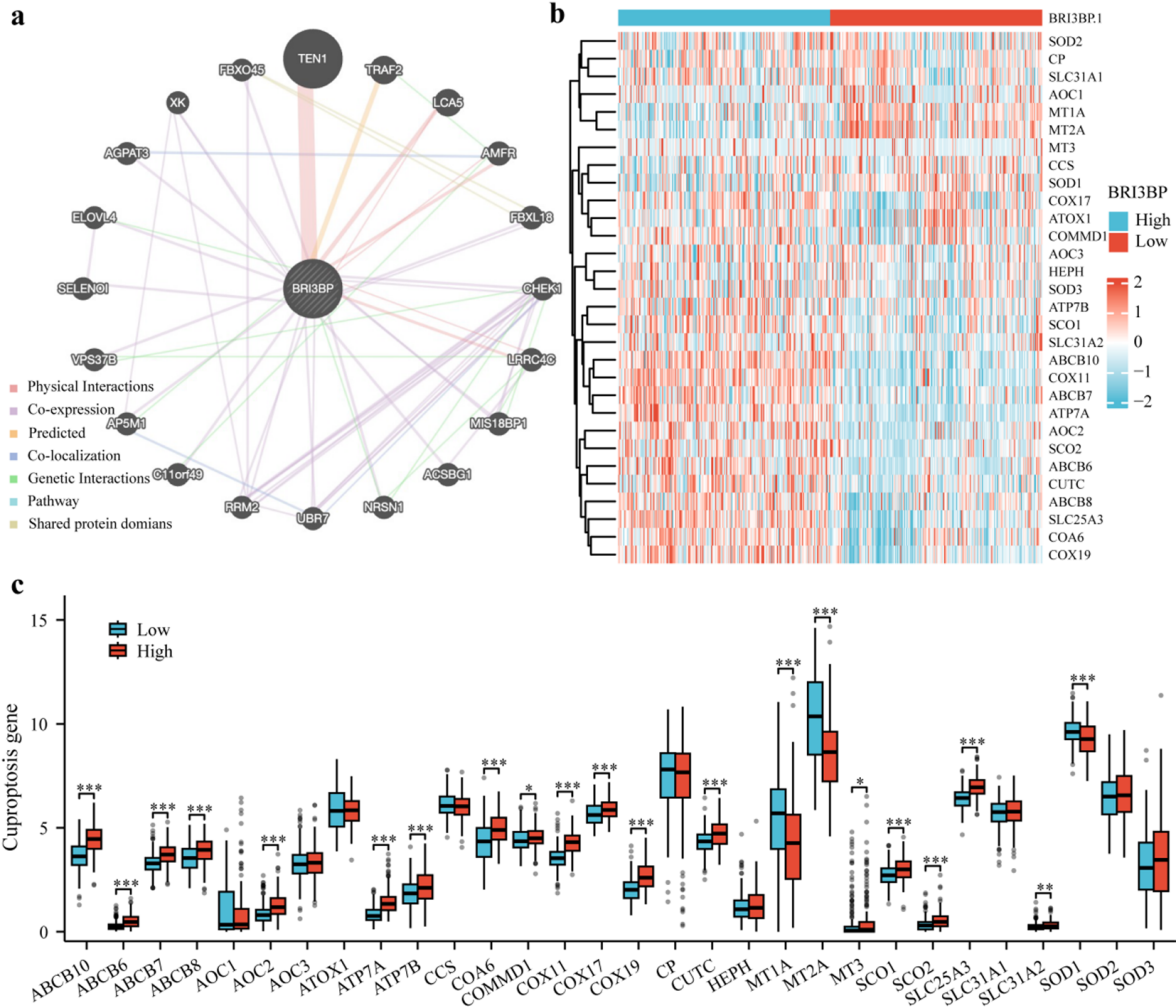
BRI3BP-related genes functional analysis and Correlation genes in cuproptosis pathway. (**a**) GeneMANIA Gene Interaction Network related to BRI3BP. (**b**) Heat map of the cuproptosis-related genes in different BRI3BP expression groups. (**c**) Expression of cuproptosis-related genes in the different BRI3BP expression groups. ns: *P* ≥ 0.05; *P < 0.05; **P < 0.01; ***P < 0.001.

To clarify the functional landscape of genes associated with BRI3BP, we performed an interactome analysis with GeneMANIA, which integrates heterogeneous genomic and proteomic datasets to predict functional gene networks (Fig. 5a). Enrichment analysis highlighted a strong association between BRI3BP and copper homeostasis—a process intimately linked to cuproptosis. Given the recent recognition of cuproptosis, a copper-dependent form of programmed cell death, as a pivotal mechanism in HCC, we next explored the relationship between BRI3BP expression and a panel of cuproptosis-related genes in the TCGA cohort. Correlation analysis across 30 such genes uncovered a complex regulatory network. BRI3BP expression showed pronounced positive correlations with key regulators of copper handling and transport, including ABCB10, ABCB6, ABCB7, ABCB8, AOC1, AOC2, ATP7A, ATP7B, COA6, COMMD1, COX11, COX17, COX19, CUTC, MT3, SCO1, SCO2, SLC25A3, and SLC31A2, while exhibiting inverse correlations with MT1A, MT2A, and SOD1 (Fig. 5b, c). These findings suggest that BRI3BP modulates copper-dependent cellular processes through a multifaceted regulatory network.

### Genetic alterations and DNA methylation of BRI3BP in HCC

**Fig. 6:**
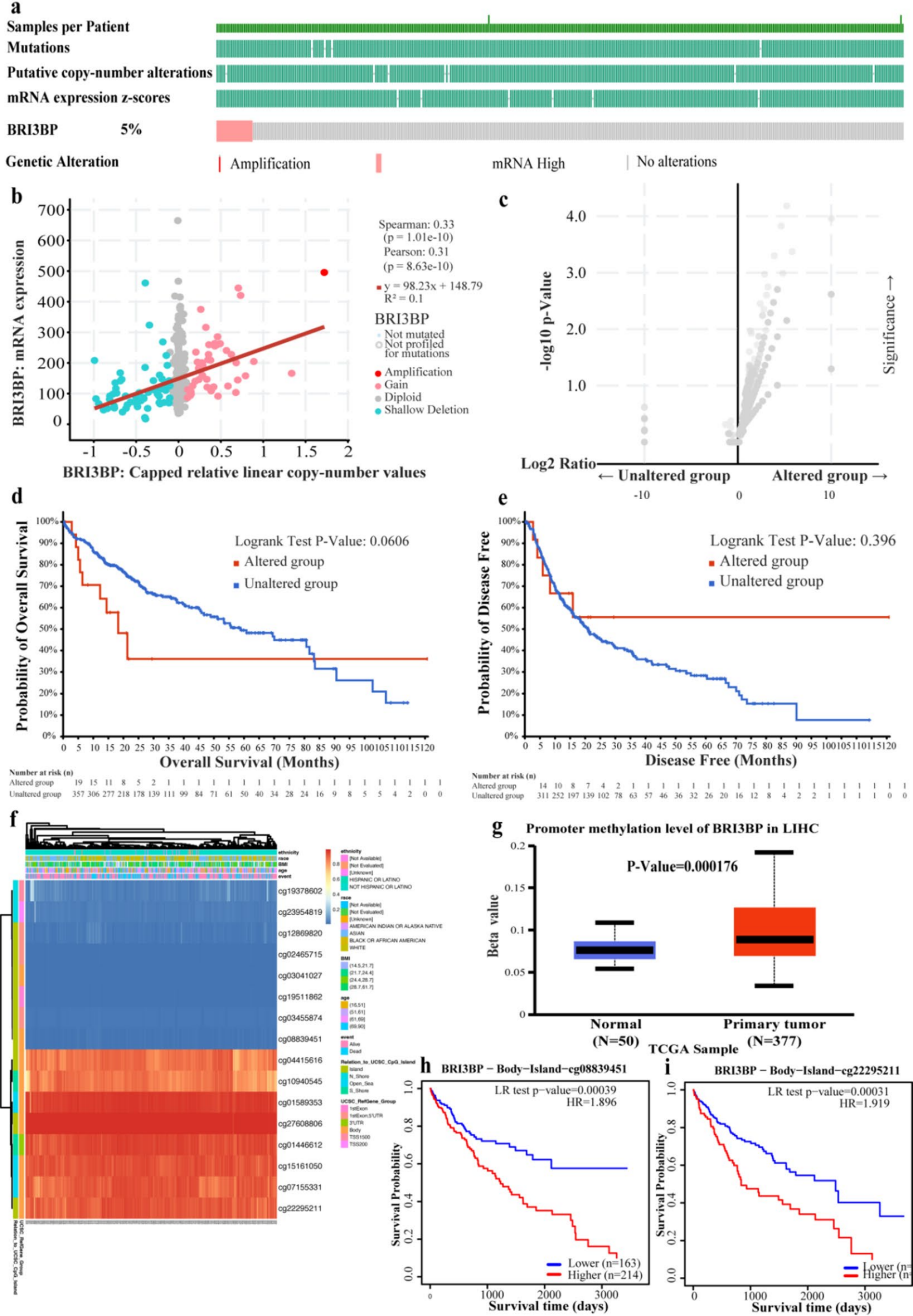
Mutations and DNA methylation levels of BRI3BP and the prognosis analysis in HCC. (**a**) Mutation levels of the BRI3BP in cBioPortal. (**b**, **c**) The correlation between *BRI3BP* mRNA and gene mutation. (**d**) Relationship between BRI3BP gene mutation and OS in HCC. (**e**) Relationship between BRI3BP gene mutation and DFS in HCC. (**f**) Correlation between *BRI3BP* mRNA expression and methylation level from the MethSurv database. (**g**) BRI3BP methylation levels from the UALCAN database. (**h**, **i**) Prognostic value of methylation at specific CpG sites: cg08839451 (**h**) and cg22295211 (**i**) (OS analysis).

To elucidate the molecular mechanisms governing BRI3BP upregulation in HCC, we conducted a comprehensive analysis of genetic and epigenetic alterations using multiple bioinformatics platforms. Analysis through cBioPortal revealed that BRI3BP genetic alterations, predominantly comprising mutations and amplifications, were present in approximately 5% of HCC cases (Fig. 6a and c). Intriguingly, Kaplan-Meier survival analyses demonstrated that these genetic alterations did not significantly impact OS (*P* = 0.0606) or DFS (*P =* 0.396) (Fig. 6d and e). Notably, our methylation profiling revealed a striking difference in BRI3BP promoter methylation status between HCC and normal liver tissues, with significantly elevated methylation levels observed in malignant tissues (*P* = 0.000176; Fig. 6g). Further exploration in the MethSurv database uncovered hypermethylation of specific CpG sites (Fig. 6f), such as cg08839451 and cg22295211(Fig. 6h and i), which were linked to poorer prognosis. These results suggest that epigenetic changes, rather than genetic mutations, may significantly contribute to abnormal BRI3BP expression in HCC and its impact on patient outcomes.

### Association between BRI3BP expression and immune infiltration in HCC

**Fig. 7:**
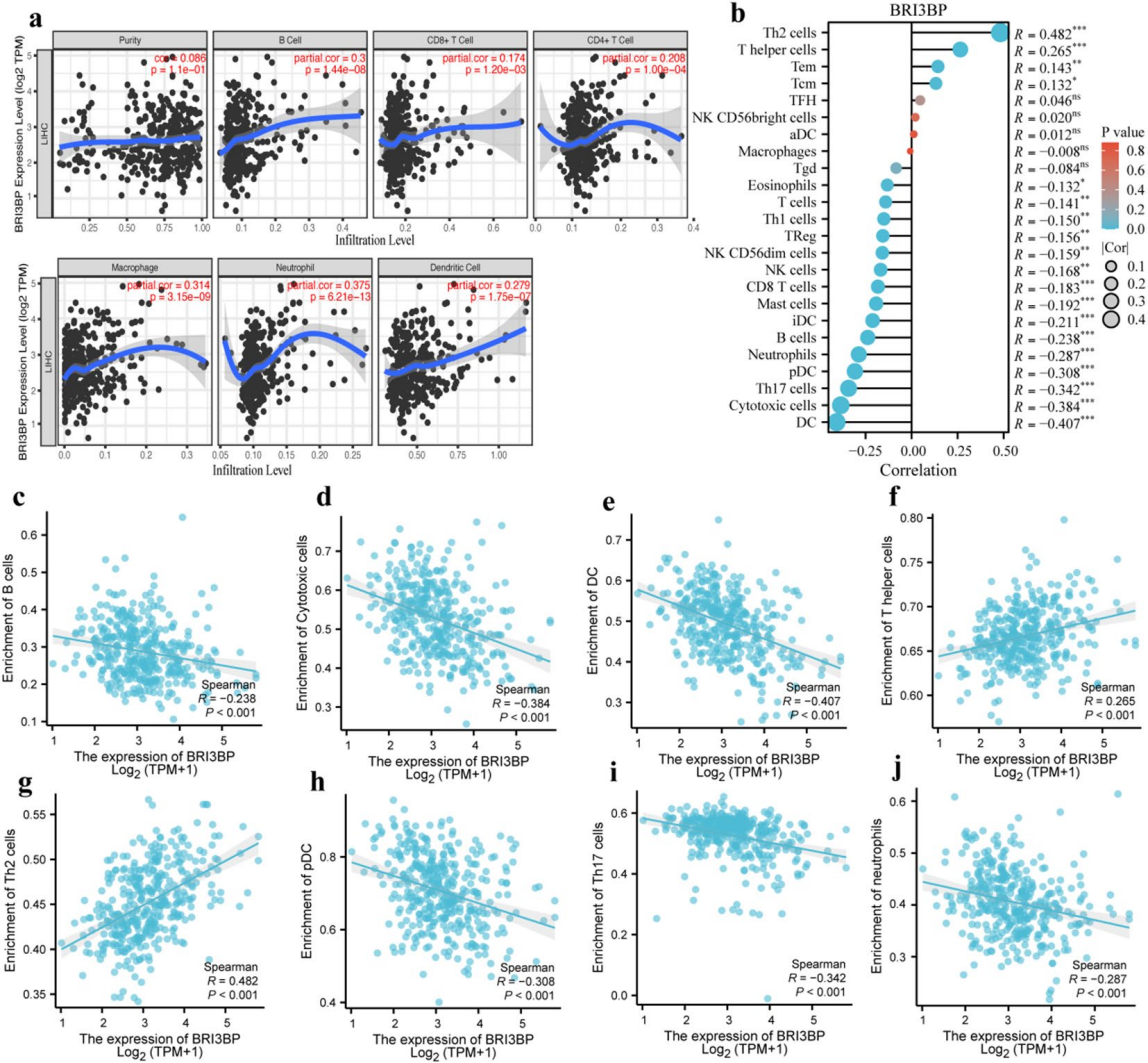
Correlation of BRI3BP expression with tumor microenvironment. (**a**) Correlation of BRI3BP with tumor purity and immune cell infiltration status in the TIMER database. (**b**) Bubble plot showing associations between BRI3BP and immune cells. (**c**–**j**) Scatter plots of correlations between BRI3BP expression and B cells (**c**), cytotoxic cells (**d**), dendritic cells (**e**), T helper cells (**f**), Th2 cells (**g**), plasmacytoid dendritic cells (**h**), Th17 cells (**i**), and neutrophils (**j**). ns: *P* ≥ 0.05; *P < 0.05; **P < 0.01; ***P < 0.001.

To delineate BRI3BP’s role in shaping the HCC immune microenvironment, we employed TIMER 2.0, which revealed a strong positive correlation between BRI3BP expression and immunosuppressive infiltrates. Specifically, we observed correlations with CD4 + T cells (*r* = 0.208, *P* = 1.00e-04), B cells (*r* = 0.3, *P* = 1.44e-08), neutrophils (*r* = 0.375, *P* = 6.21e-13), CD8 + T cells (*r* = 0.174, *P* = 1.20e-03), dendritic cells (DC; *r* = 0.279, *P* = 1.75e-07), as well as macrophages (*r* = 0.314, *P* = 3.15e-09; Fig. 7a). Furthermore, we utilized the ssGSEA algorithm to investigate the association between BRI3BP expression and 24 distinct categories of intra-tumoral immune cells. Significant positive correlations were found between BRI3BP expression and Th2 cells (*r* = 0.482, *P* < 0.001), T helper cells (*r* = 0.265, *P* < 0.001), central memory T cells (Tcm) (*r* = 0.132, *P* < 0.01) and effector memory T cells (Tem) (*r* = 0.143, *P* < 0.05; Fig. 7b). Conversely, BRI3BP expression showed significant negative correlations with several immune-cell subsets, including DC (*r* = − 0.407, *P* < 0.001), Th17 cells (*r* = − 0.342, *P* < 0.001), neutrophils (*r* = − 0.287, *P* < 0.001), plasmacytoid DC (*r = −* 0.308, *P <* 0.001), and cytotoxic cells (*r = −* 0.384, *P* < 0.001; Fig. 7c–j). Scatter plots illustrate these relationships for B cells, cytotoxic cells, conventional and plasmacytoid DC, T-helper cells, Th2 cells, Th17 cells, and neutrophils (Fig. 7c–j). To validate these findings, we performed IHC on 10 HCC samples. High BRI3BP expression was associated with lower CD8 + T cell infiltration and higher CD68 + macrophage density (Supplementary Fig. S2b), supporting an immunosuppressive role. Collectively, the results indicate a complex interplay between BRI3BP and the HCC immune microenvironment, suggesting that BRI3BP may mediate immune evasion or modulation in this cancer.

### Interaction network of BRI3BP and prediction of targeted drug therapy outcomes

**Fig. 8:**
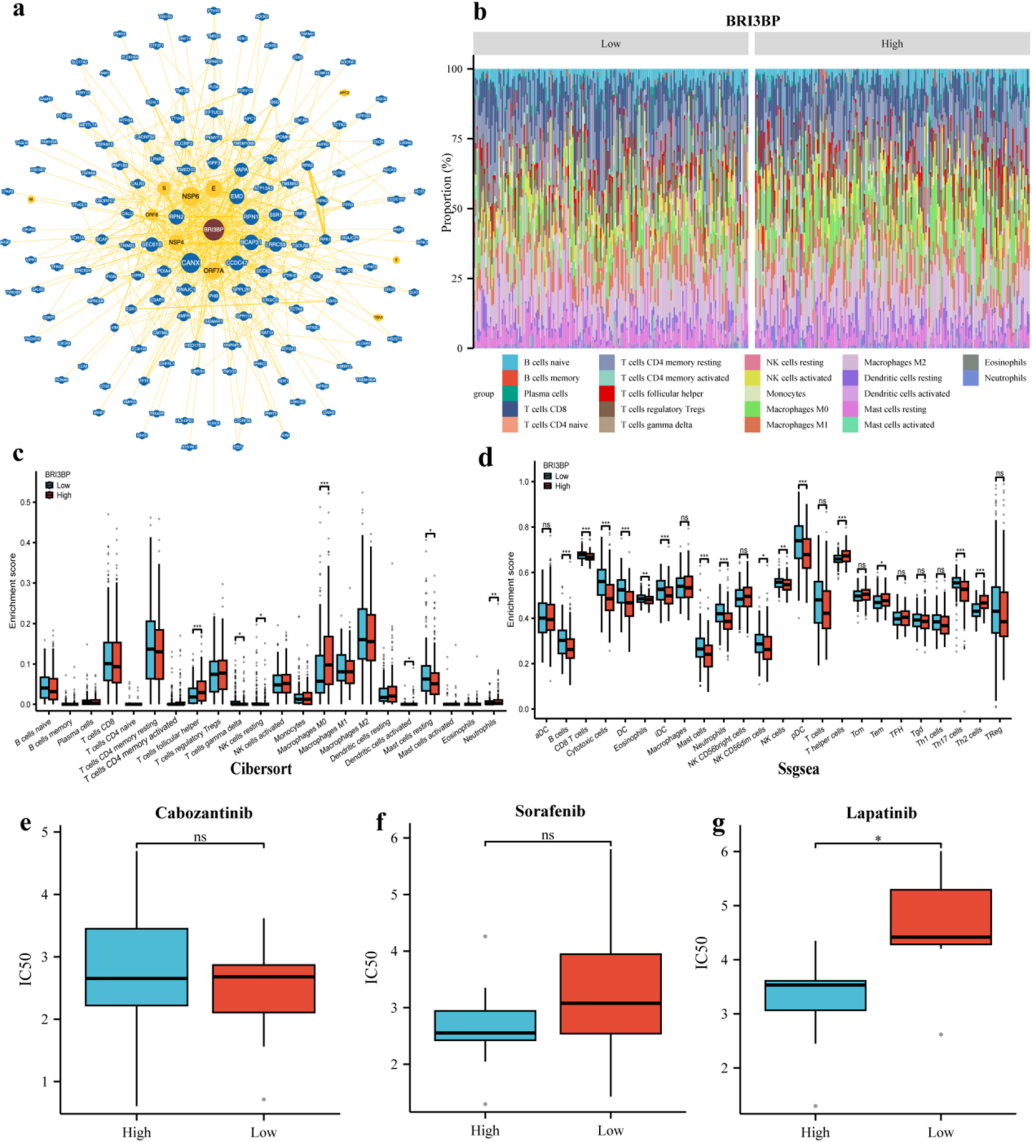
Interaction network of BRI3BP and prediction of targeted drug therapy. (**a**) Interaction network of BRI3BP from the BioGRID database. (**b**) Stacked Bar Chart of 22 immune cells and BRI3BP from CIBERSORT. The differences in immune cell infiltration levels between the high and low BRI3BP expression groups from CIBERSORT (**c**) or Ssgsea (**d**). (**e**–**g**) IC50 values of cabozantinib (**e**), sorafenib (**f**), and lapatinib (**g**) in HCC patients with low BRI3BP expression (orange bars) and high BRI3BP expression (blue bars). ns: *P* ≥ 0.05; *P < 0.05; **P < 0.01; ***P < 0.001.

To explore the broader functional context of BRI3BP, we built a protein-interaction network using BioGRID, which integrates gene–gene interactions, post-translational modifications, and small-molecule associations. The analysis identified strong links between BRI3BP and ORF7A, NSP6, NSP4, RPN1, RPN2, CANX, CCDC47, BCAP31, EMD, LRRC59, and SEC61B (Fig. 8a), implying that BRI3BP participates in diverse cellular processes through these partners.

We next quantified immune infiltration with complementary deconvolution approaches (CIBERSORT and single-sample GSEA). BRI3BP expression correlated significantly with follicular helper T cells, Th2 cells, cytotoxic cells, DC, mast cells, CD8 + T cells, neutrophils, immature DC, Th17 cells, plasmacytoid DC, B cells, and T-helper cells (Fig. 8b–d), further highlighting its immunomodulatory role.

Drug-sensitivity analysis revealed that tumors with low BRI3BP expression displayed a higher half-maximal inhibitory concentration (IC50) for lapatinib, indicating reduced susceptibility, whereas the sensitivities to cabozantinib and sorafenib were comparable between the low- and high-expression groups (Fig. 8e–g). Experimental validation in HepG2 cells showed that BRI3BP overexpression decreased the IC50 for lapatinib (*P* < 0.05), while knockdown increased it (*P* < 0.05) (Supplementary Fig. S2c). Consistently, BRI3BP overexpression in Huh7 cells upregulated ROCK1, ROCK2, PDGFB, and p-MYTP1, whereas knockdown of BRI3BP in HepG2 cells reduced their abundance (Supplementary Fig. S3), suggesting the activation of the ROCK pathway. This pathway-specific modulation may underlie the observed differences in sensitivity to targeted agents, such as lapatinib.

These findings indicate that BRI3BP influences immune modulation within the tumor microenvironment, thereby affecting tumor progression and therapeutic response. We conducted a comprehensive analysis of its association with drug response profiles, focusing specifically on clinically approved targeted therapies for HCC, such as cabozantinib, sorafenib, and lapatinib. Notably, our analysis revealed a higher IC50 value for lapatinib in the low BRI3BP expression group, indicating reduced sensitivity to this agent among patients with low BRI3BP expression (Fig. 8e and g). Thus, BRI3BP is a potential predictive biomarker for drug sensitivity, which could uncover essential insights for optimizing therapeutic strategies in HCC.

## Discussion

HCC remains a global health burden with escalating incidence and 5-year survival rates below 20%^17^. While surgical resection represents the gold standard therapeutic intervention for early-stage disease, the clinical landscape is significantly complicated by the remarkably high post-hepatectomy recurrence rates, with more than 50% of patients experiencing disease relapse within a five-year window post-surgery^18–20^. The current limitations in HCC prognostication and treatment highlight the critical imperative to identify robust molecular biomarkers that can serve as complementary tools to aid in therapeutic decision-making. The discovery of such biomarkers has the potential to advance personalized treatment approaches and improve the clinical management of HCC, ultimately leading to better patient outcomes.

Our study provides the first multi-omic dissection of BRI3BP expression with the clinical characteristics in HCC. Our study revealed significant associations between BRI3BP expression and key clinical parameters—namely, vascular invasion and elevated α-fetoprotein (AFP, a serum biomarker for HCC) levels. Functional assays substantiated these findings: BRI3BP overexpression markedly enhanced migration and invasion in HCC cell lines. Comprehensive survival modeling further validated the utility of BRI3BP. Kaplan–Meier curves and multivariate Cox regression showed that high BRI3BP expression independently predicted shorter OS, DFS, and PFI. Time-dependent ROC analysis demonstrated strong discriminative capacity, while calibration plots exhibited minimal deviation between predicted and observed survival probabilities. The C-index for the full nomogram (BRI3BP + T stage + tumor status) was 0.657 (95% CI: 0.628–0.687), representing a meaningful improvement over the model with T stage and tumor status alone (C-index: 0.602; 95% CI: 0.579–0.625). This improvement is clinically relevant because it demonstrates that BRI3BP adds significant prognostic power beyond standard tools, potentially enabling better risk stratification and treatment decisions for HCC patients. Mechanistically, BRI3BP activated the ROCK signaling cascade and promoted HCC-cell motility, implicating this pathway in tumor progression. Together, these data highlight BRI3BP’s value as a prognostic biomarker and suggest it as a candidate therapeutic target—particularly for strategies aimed at the ROCK pathway^21^.

Additionally, TCGA datasets were analyzed to reveal distinct transcriptional profiles associated with BRI3BP expression levels. We identified 247 DEGs with a marked asymmetry in expression patterns: 218 genes showing significant upregulation and 29 exhibiting downregulation in BRI3BP-high samples. GO and KEGG pathway enrichment analyses were conducted to characterize the functional interplay among the DEGs. The results indicated significant enrichment in pathways related to channel activity, apical plasma membrane localization, and stress responses triggered by copper ions. Cuproptosis, characterized as a copper-dependent mechanism of regulated cellular demise, has emerged as a crucial pathway in HCC and represents a promising target for therapeutic intervention^22–24^. In our research, we investigated the relationship between the expression of BRI3BP and 30 genes associated with cuproptosis, uncovering the potential role of BRI3BP in processes related to cuproptosis, which may impact treatment outcomes in HCC patients. The progression of cancer is intricately tied to genetic mutations and the methylation of DNA^25–27^. Our findings indicated that the mutation frequency of BRI3BP in HCC was 5%. Additionally, the analysis of methylation patterns demonstrated that BRI3BP exhibited elevated methylation levels in HCC tissues compared to adjacent non-tumor liver tissues. Further exploration using the MethSurv database revealed hypermethylation of specific CpG sites, such as cg08839451 and cg22295211, which were associated with a poorer prognosis.

Immunohistochemistry confirmed that high BRI3BP tumors contain fewer CD8 + T cells and more CD68 + macrophages, in line with the bioinformatics data. These findings are consistent with the notion that BRI3BP may contribute to an immunosuppressive tumor microenvironment. The unexpected finding that BRI3BP overexpression lowers lapatinib IC50 is consistent with earlier reports that ROCK-dependent cytoskeletal stress sensitizes hepatoma cells to EGFR inhibition^28–30^. Although ROCK signaling is a well-established driver of HCC metastasis and chemoresistance, our data reveal a counterintuitive, pathway-specific vulnerability: BRI3BP-high/ROCK-activated cells exhibit heightened sensitivity specifically to the EGFR inhibitor lapatinib. This apparent paradox, where high BRI3BP predicts aggressive disease yet increased lapatinib sensitivity, may be explained by a model wherein ROCK activation induces a state of compensatory dependency on EGFR signaling, a concept supported by prior oncogenic addiction paradigms^28^. This suggests that BRI3BP-driven aggressiveness is coupled to a therapeutically actionable liability. Thus, while prognostic for poor survival, high BRI3BP expression may also predict a favorable response to EGFR-targeted therapies, identifying a patient subgroup with aggressive disease that could potentially benefit from lapatinib.

Metabolic reprogramming within the tumor microenvironment is a defining characteristic of both cancer cells and T lymphocytes. CD4 + T cells, which belong to the T helper cell subset, serve as critical indicators of the immune status of an organism^31–34^. Notably, our observations revealed a significant positive correlation between the expression of BRI3BP and the prevalence of specific immune cell populations, such as CD8 + T cells and B cells. BRI3BP expression correlated strongly with immunosuppressive subsets—Th2 cells (*r* = 0.482, *P* < 0.001) and CD4 + T cells (*r* = 0.208, *P* = 1.00e-04), indicating that BRI3BP may foster an immunosuppressive tumor microenvironment linked to resistance to immune-checkpoint blockade in HCC^35^. Accordingly, BRI3BP emerges as a candidate target for modulating immunotherapy response. Consistently, HCC cells with high BRI3BP expression showed greater sensitivity to small-molecule inhibitors—exemplified by lower IC50 values for lapatinib—than their low-expression counterparts.

The analysis of drug sensitivity regarding BRI3BP and lapatinib showed encouraging results; nonetheless, additional investigations are necessary to validate these effects. Furthermore, we propose the creation of a prognostic nomogram that combines BRI3BP expression with essential clinical factors, providing a practical tool for assessing individual risk. This nomogram would provide a quantitative means of predicting patient outcomes, enabling healthcare providers to make informed treatment decisions. Overall, this study found a close association between BRI3BP and HCC. This association will not only contribute to elucidating the pathogenesis of HCC but will also identify prognostic markers and therapeutic targets for HCC.

Several study limitations warrant consideration. First, the conclusions rely predominantly on bioinformatics analyses and, therefore, lack the experimental validation required to confirm BRI3BP’s functional contribution to HCC progression. Second, dependence on publicly available datasets may introduce bias through limited sample size or incomplete representation of the broader HCC population, which could constrain the generalizability of the findings. Third, the absence of prospective clinical validation studies limits the immediate translational applicability of the results. Due to access constraints, we used clinical samples from healthy donors instead of primary human hepatocytes (PHH), which may have affected the accuracy of the reference data. These constraints emphasize the need for future investigations that integrate both laboratory experimentation and clinical research to verify and expand the current observations. Furthermore, the observed association between high BRI3BP expression and poor tumor differentiation could potentially confound the results of the pathway enrichment analyses, particularly for metabolic processes.

## Conclusions

This comprehensive investigation identifies BRI3BP as a clinically significant molecular factor in HCC: the gene is markedly overexpressed and closely associated with adverse outcomes. Experimental data indicate that BRI3BP enhances cell migration and invasion and activates the ROCK pathway, highlighting its multifaceted role in tumor biology. Collectively, the analyses demonstrate that BRI3BP offers robust prognostic discrimination and may predict sensitivity to targeted agents such as lapatinib in preclinical models, supporting its candidacy as a prognostic biomarker and potential therapeutic target in HCC—pending further experimental confirmation.

## Methods

### Data sources and specimen collection

Transcriptomic profiling data and clinical annotations for HCC were retrieved from the TCGA cohort. Gene expression microarray datasets (GSE62232, GSE121248, GSE76427, and GSE84005) were curated from the Gene Expression Omnibus (GEO) database, ensuring comprehensive multi-cohort validation. Fresh-frozen HCC tissues and matched adjacent non-tumorous specimens were prospectively collected at the Hangzhou First People’s Hospital, affiliated with the Medical School of Westlake University. The study was approved by the hospital’s Ethics Committee (No. 2025ZN044-1) in full compliance with the Declaration of Helsinki. Written informed consent was obtained from all patients before tissue acquisition.

### Correlation analysis between BRI3BP expression and clinicopathological parameters

The clinicopathological relevance of BRI3BP in HCC was assessed by integrating mRNA expression profiles from TCGA with detailed phenotypic annotations. We evaluated the associations between BRI3BP expression (stratified by median cutoff) and key parameters, including pathological stage (AJCC 9th edition), histological grade (Edmondson-Steiner classification), tumor status, serum alpha-fetoprotein (AFP) levels, and vascular invasion. The prognostic utility was further quantified using Kaplan-Meier analysis for overall survival (OS), disease-free survival (DFS), and progression-free interval (PFI), followed by univariate Cox regression analysis.

### Construction and validation of prognostic nomogram

A clinical prognostic nomogram incorporating BRI3BP-derived risk scores, tumor stage, and vascular invasion status was developed using the R “rms” package to predict 1-, 3-, and 5-year OS probabilities in the TCGA cohort. Model calibration was performed via 1,000 bootstrap resamples to compare predicted versus observed survival rates. The predictive performance was assessed using time-dependent ROC analyses via the timeROC package, and model discrimination was further quantified by calculating Harrell’s concordance index (C-index). Multivariate Cox proportional hazards regression confirmed the independence of BRI3BP-associated risk scores from conventional prognostic variables.

### Differentially expressed gene analysis

Participants were categorized into two distinct cohorts based on the median expression levels of BRI3BP observed in the TCGA_LIHC dataset: individuals with elevated BRI3BP expression and those with diminished expression. To detect differentially expressed genes (DEGs), the ‘DESeq2’ package within the R programming environment was utilized, applying the specific criteria of |log2FC| ≥ 2 and an FDR < 0.05. Additionally, a targeted Spearman’s rank-order correlation analysis was used to quantitatively assess the covariation between BRI3BP transcript levels and the five top-ranked upregulated/downregulated DEGs, stratified by their statistical significance. The results were depicted in volcano plots to effectively highlight the significant DEGs.

### Functional enrichment analysis, mutation, and DNA methylation

To dissect the molecular networks that drive BRI3BP-mediated hepatocarcinogenesis, we performed a multi-tiered functional genomic interrogation.Functional enrichment analysis was evaluated in three complementary dimensions: (i) Gene Ontology (GO) annotation of biological process, cellular component, and molecular function; (ii) Kyoto Encyclopedia of Genes and Genomes (KEGG) pathway mapping; and (iii) Gene Set Enrichment Analysis (GSEA) using the Molecular Signatures Database (MSigDB) c2.cp.all.v2022.1.Hs.symbols.gmt collection. Analyses were implemented with the clusterProfiler (v4.6.2) and org.Hs.eg.db (v3.16.0) R packages, adopting an FDR < 5%. Furthermore, BRI3BP-mutated samples were explored in cBioPortalDNA to catalog somatic alterations. Bisulphite-sequencing-derived promoter-methylation data from UALCAN (TCGA-HCC cohort) were coupled with MethSurv-based modeling to relate CpG-island methylation to overall survival, delineating methylation–epigenetic crosstalk.

### Gene interaction network analysis

Gene interaction networks were constructed to clarify BRI3BP’s functional context. BioGRID data were analyzed to identify drug targets and to map physical, genetic, chemical, and high-throughput interactions. A protein–protein interaction network of BRI3BP-associated proteins was also generated with GeneMANIA. These network-level analyses illuminate the molecular interactions and potential functional roles of BRI3BP in HCC.

### Immune infiltration analysis associated with BRI3BP expression

Enrichment scores for 24 distinct immune cell types were quantified using the single-sample Gene Set Enrichment Analysis (ssGSEA) algorithm implemented within the ‘gsva’ R package. To further validate these findings, we employed TIMER 2.0 (http://timer.cistrome.org/) to assess the infiltration levels of key immune cells. Subsequently, TCGA samples were stratified based on BRI3BP expression levels (high versus low), and comparative analyses of immune cell enrichment scores were performed to delineate the impact of BRI3BP expression on the HCC immune microenvironment.

### Targeted drug therapy outcome prediction

To evaluate the predicted efficacy of targeted therapies in HCC patients, we sourced pharmacogenomic data from the Genomics of Drug Sensitivity in Cancer (GDSC) database (https://www.cancerrxgene.org). Subsequently, we employed the ‘oncoPredict’ R package to derive the half-maximal inhibitory concentration (IC50) values for cabozantinib, sorafenib, and lapatinib. This approach enabled us to investigate the potential association between varying levels of BRI3BP expression and the therapeutic response to these agents.

### Quantitative real-time PCR

Total RNA was isolated from cells using a TRIzol^®^ kit (Thermo Fisher Scientific, USA), and RNA concentration was determined. cDNA was synthesized using the PrimeScript™RT kit (TAKARA, Japan). qRT-PCR was performed using the Sybr Premix Ex Taq II kit (TAKARA, Japan) and the ABI7500 detection system (Applied Biosystems, USA). β-Actin was used as the reference. The primer sequences employed are listed in Table 3.

**Table 3:** qRT-PCR primer sequences.

Gene	Primer sequence(5’→3’)
BRI3BP-F	CTGGGAGTGGATATGTTCGTG
BRI3BP-R	CTGGGCTGAAATACTGGGAC
β-Actin-F	ATGTGGCCGAGGACTTTGATT
β-Actin-R	AGTGGGGTGGCTTTTAGGATG

### Western blot

Protein extracts were prepared by lysing samples from each group in RIPA buffer (Beyotime, China) for 10 min, and then the supernatant was obtained. Proteins were resolved on 10% SDS-PAGE gels (Solaibao, China). Target protein bands were excised from the gels and transferred onto PVDF membranes (Shanghai Shenggong, China). The membranes were blocked and washed with primary antibodies overnight at 4 °C and then incubated with diluted secondary antibodies for 2 h at room temperature. Finally, immunoreactive bands were visualized using an ECL detection kit (Bio-Rad, USA) on an iBright imaging system (Thermo Fisher, USA). In this study, anti-BRI3BP (Cusabio, China), anti-p-MYTP1 (MilliporeSigma), and anti-MYTP1 (Cell Signaling Technology) served as the primary antibodies.

### Cell culture and BRI3BP modulation

HepG2 and Huh7 (HCC) cell lines (from the Cell Bank of the Chinese Academy of Sciences, Shanghai) were cultured in DMEM (Gibco) supplemented with 10% fetal bovine serum (FBS) and 1% penicillin–streptomycin at 37 °C in 5% CO2. For BRI3BP overexpression, Huh-7 cells were transfected with pcDNA3.1-BRI3BP or the empty pcDNA3.1 vector (GenScript) using Lipofectamine 3000 (Invitrogen). For knockdown, HepG2 cells were treated with BRI3BP-specific siRNA (si-BRI3BP, GenePharma, Table S1) or non-targeting control siRNA (si-NC) using Lipofectamine RNAiMAX (Invitrogen). Transfection efficiency was verified 48 h later by qRT-PCRs. For functional assays we used HepG2 cells in both over-expression and siRNA experiments.

### Transwell migration and invasion assays

HCC cells with BRI3BP modulation were assessed in Transwell chambers with 8 μm pores (Corning).*Migration*: 5 × 10^4^ cells in 200 µL serum-free DMEM were placed in the upper chamber; 600 µL DMEM containing 10% FBS was added to the lower well. For invasion, chambers were pre-coated with Matrigel (1:8 in SFM; BD Biosciences), and 5 × 10^4^ cells were seeded as above. After 24 h, non-migrated or non-invaded cells were removed. Cells on the underside were fixed with 4% PFA, stained with 0.1% crystal violet, and counted in five random fields at 200 × magnification (Olympus).

### Immunohistochemsitry(IHC)

We performed immunohistochemistry on formalin-fixed paraffin-embedded (FFPE) sections from five high- and five low-BRI3BP HCC tumors. After standard deparaffinization and rehydration, antigen retrieval was achieved with citrate buffer (pH 6.0). We then blocked endogenous peroxidase activity with 3% H_2_O_2_. Sections were probed with primary antibodies targeting BRI3BP (Sigma HPA042524), CD8 (Abcam ab178089), and CD68 (Abcam ab213363) in a 4 °C overnight incubation. Following application of a secondary antibody, staining was developed using DAB, and nuclei were counterstained with hematoxylin. A blinded histological evaluation was conducted by two independent pathologists to ensure objective scoring.

### Drug sensitivity assay(MTT)

HepG2 cells were transfected with BRI3BP constructs or controls for 48 h, then treated with lapatinib (0–10 µM) for 48 h. Cell viability was measured using the MTT assay (490 nm absorbance after DMSO solubilization). IC50 values were calculated via nonlinear regression (GraphPad Prism 9.0). All experiments were performed in triplicate.

### Statistical analyses

BRI3BP expression across tissue types was compared with a two-sample t-test; additional comparisons employed the Wilcoxon rank-sum test and logistic regression. The prognostic impact was evaluated using Kaplan–Meier curves with log-rank testing. *BRI3BP* mRNA levels in LO2, HepG2, and Huh7 cells were analyzed by one-way ANOVA followed by Tukey’s post-hoc test. Migration and invasion data (BRI3BP-overexpression or knockdown groups versus control) were analyzed with two-sample t-tests. All analyses were performed in R v4.2.1 or GraphPad Prism v9.0.

## Supplementary Information

Below is the link to the electronic supplementary material.

Supplementary Material 1

## Data Availability

The TCGA and GEO data are available in a public, open-access repository. Other data related to patients that support the findings of this study are not publicly available due to privacy reasons but are available from the corresponding author upon reasonable request.
