# Supplementary material for: Multi-omic analysis reveals elevated BRI3BP expression associated with hepatocellular carcinoma progression and poor prognosis
Source: Sci Rep. 2025 Oct 31;15:38151. doi: 10.1038/s41598-025-22072-5 (PMC12578974; doi:10.1038/s41598-025-22072-5)
Supplement: Supplementary file 1 — Supplementary Material 1 [file 41598_2025_22072_MOESM1_ESM.docx]

**Supplementary Materials**

**
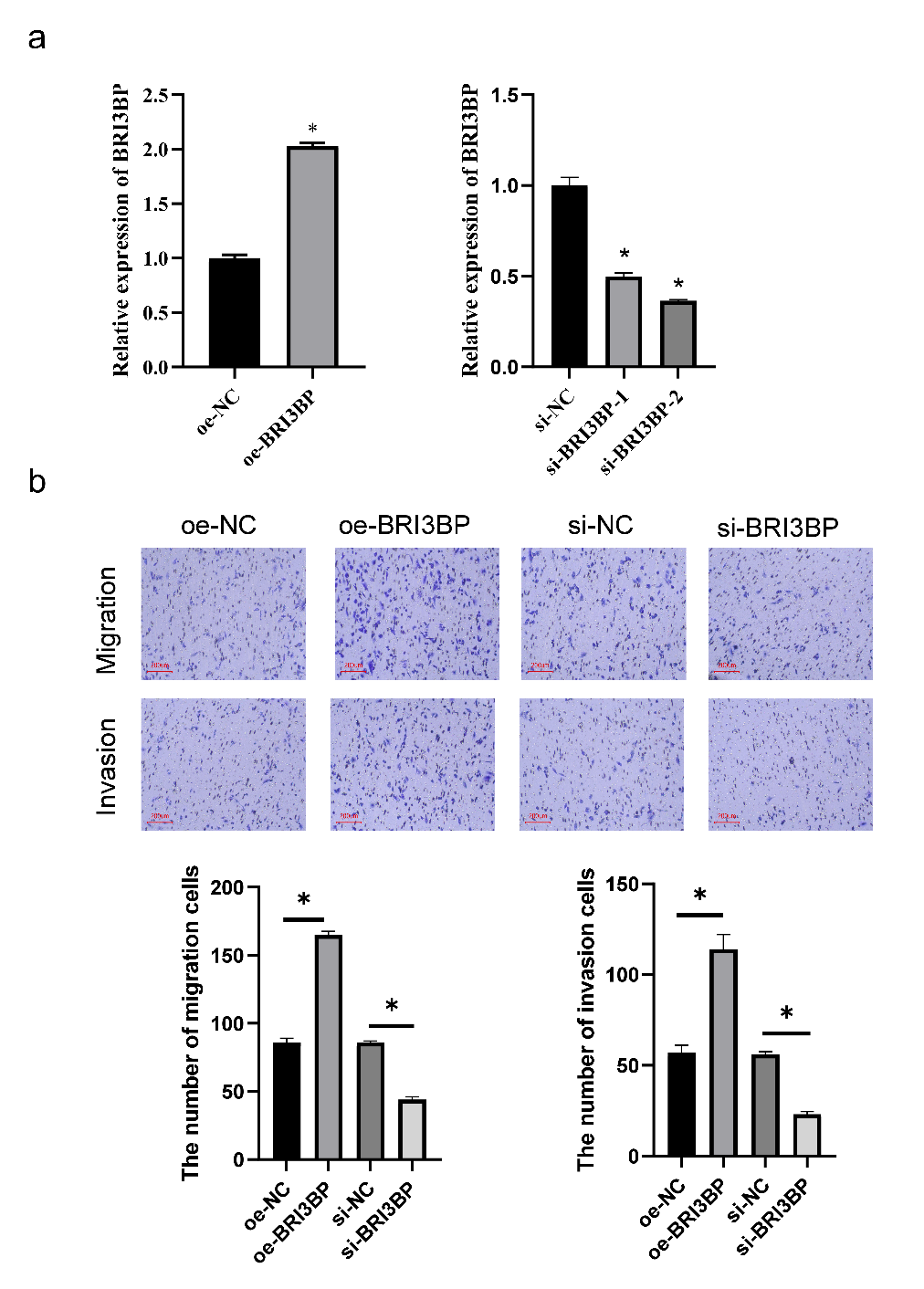
**

**Figure S1. BRI3BP Expression and Functional Assays in HCC Cells**.

(a) qRT-PCR analysis showing increased *BRI3BP* mRNA expression in Huh7 cells transfected with a BRI3BP overexpression vector compared to control vector-transfected cells, and decreased *BRI3BP* mRNA expression in HepG2 cells transfected with siRNAs targeting BRI3BP compared to control siRNA-transfected cells. (b) Transwell assay results showing increased cell migration and invasion in Huh7 cells overexpressing BRI3BP compared to control cells, and reduced migration and invasion in HepG2 cells with BRI3BP knockdown via siRNA compared to control siRNA-transfected cells. Representative images and quantification of migrated/invaded cells are shown from three independent experiments. Oe-NC: control vector; oe-BRI3BP: BRI3BP overexpression vector; si-NC: control siRNA; si- BRI3BP: siRNA targeting BRI3BP. **P* < 0.05.

**
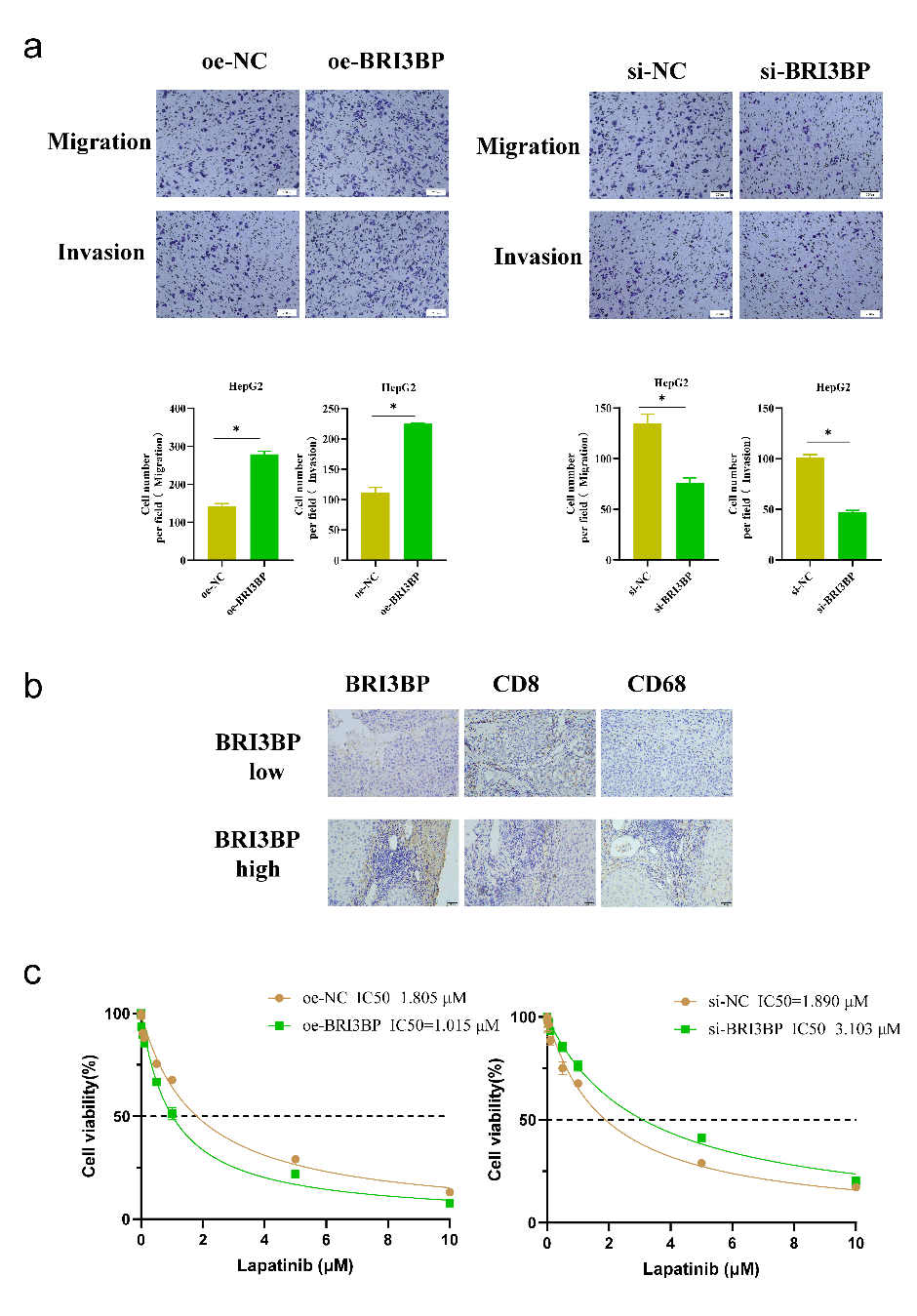
**

**Fig. S2. Experimental validation of BRI3BP’s role in HCC progression, immune modulation, and therapeutic response**.

(a) Transwell migration and invasion assays in HepG2 cells transfected with control, BRI3BP overexpression (OE), or BRI3BP knockdown (si-BRI3BP) constructs. Representative images (left) and quantitative analysis (right) show that BRI3BP OE significantly enhances cell migration and invasion, while siRNA-mediated knockdown suppresses these phenotypes. (b) Representative immunohistochemistry (IHC) images of CD8+ T cell and CD68+ macrophage infiltration in HCC tissues with high or low BRI3BP expression (n = 5 per group). Scale bars: 50 µm. High BRI3BP expression correlates with reduced CD8+ T cell density and increased CD68+ macrophage infiltration. (c) Lapatinib sensitivity in HepG2 cells with modulated BRI3BP expression. IC50 values for lapatinib were significantly lower in BRI3BP-OE cells and higher in si-BRI3BP cells compared to controls, indicating that BRI3BP expression enhances sensitivity to lapatinib. Data are presented as mean ± SD; n = 3 independent experiments. **P* < 0.05.

**
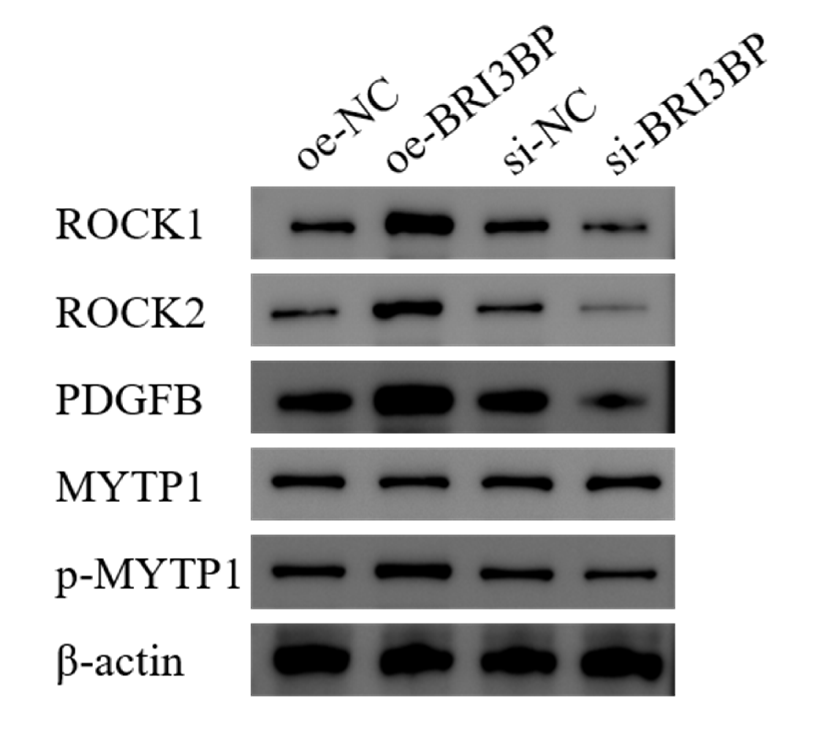
**

**Figure S3. Western Blot Analysis of ROCK Signaling Pathway Components in HCC Cells**. Western blot analysis demonstrating increased protein expression of ROCK1, ROCK2, and PDGFB, p-MYTP1, and MYTP1 in Huh7 cells transfected with a BRI3BP overexpression vector compared to control vector-transfected cells, and decreased expression of ROCK1, ROCK2, PDGFB, p-MYTP1, and MYTP1 in HepG2 cells transfected with siRNA targeting BRI3BP compared to control siRNA-transfected cells. β-actin was used as a loading control. Oe-NC: control vector; oe-BRI3BP: BRI3BP overexpression vector; si-NC: control siRNA; si- BRI3BP: siRNA targeting BRI3BP.

| **Table S1 siRNA Primers** | |
| --- | --- |
| siRNA | sequence |
| siRNA- BRI3BP-1 | TTTCCATGTCCTGCGTGTACA |
| siRNA- BRI3BP-2 | CTCCAACCTGTCCCAGTATTT |
